# Physical activity and depression later in life: a cross sectional study in Greece

**DOI:** 10.1192/j.eurpsy.2023.1974

**Published:** 2023-07-19

**Authors:** K. Argyropoulos, F. Arampatzi, A. Argyropoulou, D. Avramidis, P. Gourzis, E. Jelastopulu

**Affiliations:** 1 Postgraduate Program “Aging and Chronic Diseases Management”, School of Medicine, University of Thessaly & Hellenic Open University, Greece, Athens; 2 School of Medicine, Univesrity of Patras, Patras, Greece; 3 Psychiatry; 4 Public Health, School of Medicine, Univesrity of Patras, Patras, Greece

## Abstract

**Introduction:**

Depression is a very prevalent mental disorder among older people. Exercise and physical activity may have beneficial effects on depressive symptoms that are comparable to those of antidepressant treatments.

**Objectives:**

The purpose of the present study was to estimate the role that physical activity plays in wellbeing of older people, as well as its association with depression.

**Methods:**

A cross-sectional study was conducted among 101 people over the age of 60, who are active members of the Open Day Care Centers (K.A.P.I) of the municipality of Serres, North Greece. An anonymous questionnaire was created to record the basic demographic data of the studied population. The Greek version of the Geriatric Depression Scale (GDS-15) was used to assess depressive symptoms in the elderly, and the (IPAQ) short edition - 7 items, was applied to evaluate the physical activity of the participants. Statistical analysis was performed with a SPSS 21.

**Results:**

According to our results, as the age of the participants increases, so does the severity of the depression according to GDS-15. In addition, depressive symptoms were associated with marital status, widows in comparison to divorced, with participants living in urban areas and with illiterates and high school graduates. Furthermore, older adults with monthly income of 1000 to 2000 Euros presented to suffer more from depression than those who had a monthly income of up to 2000 Euros (p <0.05).On the other hand, physical activity based on IPAQ was strongly associated with age, married compared to not, high level of education, living in rural areas and depression (p <0.05).

**Conclusions:**

The results of the present study may contribute to further interventions in Primary Health Care for the prevention and detection of depression among older people. Furthermore, physical exercise may be an alternative or adjunct to traditional forms of treatment in mild to moderate forms of depression later in life.

**Disclosure of Interest:**

None Declared

